# BNEMDI: A Novel MicroRNA–Drug Interaction Prediction Model Based on Multi-Source Information With a Large-Scale Biological Network

**DOI:** 10.3389/fgene.2022.919264

**Published:** 2022-07-15

**Authors:** Yong-Jian Guan, Chang-Qing Yu, Li-Ping Li, Zhu-Hong You, Zhong-Hao Ren, Jie Pan, Yue-Chao Li

**Affiliations:** ^1^ School of Information Engineering, Xijing University, Xi’an, China; ^2^ College of Grassland and Environment Sciences, Xinjiang Agricultural University, Urumqi, China; ^3^ School of Computer Science, Northwestern Polytechnical University, Xi’an, China; ^4^ Key Laboratory of Resources Biology and Biotechnology in Western China, Ministry of Education, College of Life Science, Northwest University, Xi’an, China

**Keywords:** miRNA–drug interaction, BiNE, k-mer, MACCS fingerprint, deep neural network

## Abstract

As a novel target in pharmacy, microRNA (miRNA) can regulate gene expression under specific disease conditions to produce specific proteins. To date, many researchers leveraged miRNA to reveal drug efficacy and pathogenesis at the molecular level. As we all know that conventional wet experiments suffer from many problems, including time-consuming, labor-intensity, and high cost. Thus, there is an urgent need to develop a novel computational model to facilitate the identification of miRNA–drug interactions (MDIs). In this work, we propose a novel bipartite network embedding-based method called BNEMDI to predict MDIs. First, the Bipartite Network Embedding (BiNE) algorithm is employed to learn the topological features from the network. Then, the inherent attributes of drugs and miRNAs are expressed as attribute features by MACCS fingerprints and *k*-mers. Finally, we feed these features into deep neural network (DNN) for training the prediction model. To validate the prediction ability of the BNEMDI model, we apply it to five different benchmark datasets under five-fold cross-validation, and the proposed model obtained excellent AUC values of 0.9568, 0.9420, 0.8489, 0.8774, and 0.9005 in ncDR, RNAInter, SM2miR1, SM2miR2, and SM2miR MDI datasets, respectively. To further verify the prediction performance of the BNEMDI model, we compare it with some existing powerful methods. We also compare the BiNE algorithm with several different network embedding methods. Furthermore, we carry out a case study on a common drug named 5-fluorouracil. Among the top 50 miRNAs predicted by the proposed model, there were 38 verified by the experimental literature. The comprehensive experiment results demonstrated that our method is effective and robust for predicting MDIs. In the future work, we hope that the BNEMDI model can be a reliable supplement method for the development of pharmacology and miRNA therapeutics.

## Introduction

As many previous studies have shown, RNA plays a vital role in encoding, decoding, regulation, and expression of genes (Fu, 2014). Global transcriptional analyses of the human genome proved that the quantity of non-coding RNAs (ncRNA) is much larger than protein in human cells and ncRNA is involved in the regulation of stem cell pluripotency and cell division (Cawley et al., 2004; Iyer et al., 2015). In the human genome project, the newly discovered RNA genes are far more abundant than protein genes (Bentwich et al., 2005). RNA can be divided into two classes based on the length of the RNA chain, mainly including long RNA of more than 200 nucleotides and small RNA of fewer than 200 nucleotides. MicroRNAs (miRNAs) are a kind of short endogenous non-coding RNAs with 20–25 nucleotides, which may modulate the expression of genes in post-transcription (Ambros, 2001; Bartel, 2004). MiRNAs will incompletely bind to the target genes for inhibiting the transcripts, which may truncate mRNAs but does not affect the stability of mRNAs (Jiang et al., 2009).

Despite great advances in miRNA therapeutics and the theoretical knowledge between miRNAs and diseases, most of the drug targets are proteins. In human cells, less than 15% of disease-related proteins are targets of drugs (Dixon and Stockwell, 2009). This means that drug targets, which are designed through proteins, can only act on a small proportion of the human genome. In brief, most proteins are not “druggable.” As a result, ncRNAs are increasingly considered by researchers as a potential drug target. Among them, miRNA is considered a valuable drug target because it can play a key role in gene regulation when the disease occurs. Increasing number of experiments prove that there is a strong relationship between the abnormal regulation of miRNA and human diseases. For example, the expression level of miR-205 and miR-393 are potential biomarkers of mucinous colorectal cancer and colon cancers, which will be increased when cancer occurs (Eyking et al., 2016). Bommer et al. (2007) discovered that the expression level of miR-34 will be lessened in non-small cell lung cancers (Bommer et al., 2007). If miRNA can be used as drug targets, it will be conducive to the development of drug discovery and drug repositioning (Zhang et al., 2021b).

Therefore, many recent studies focus on the miRNA-based approach as a therapeutic, one of which is targeting over-expressed miRNAs (Ishida and Selaru, 2013; Bayraktar et al., 2018; Zhang et al., 2021). Kota et al. (2009) reported that miR-26a transported by adeno-associated virus (AAV) inhibits the spread of cancer cells and activates the apoptosis of cancer cells. In the previous study, Esquela-Kerscher et al. suggested that the active expression of let-7 could suppress the proliferation of tumor cells in the mice model (Fu et al., 2021). Matboli et al. (2017) demonstrated that caffeic can effectively attenuate diabetic kidney disease in rats by downregulating the expression level of miR-133b, miR-342, and miR30a (Matboli et al., 2017).

However, detecting MDI based on the experiment is a labor-intensive and time-consuming process*. In silico*, some of the prediction methods have been proposed to infer the potential interaction between miRNAs and drugs. For example, Huang et al. proposed a computational method named GCMDR, which is based on a graph convolution neural network and explores the link between miRNA and drug resistance (Ya et al., 2020). In detail, they constructed a bipartite graph integrating the fingerprint of drug compounds and miRNA functional similarity. Moreover, they learned from the idea of auto-encoder, in which they built a graph convolution-based encoder to generate the embeddings of nods and a decoder to complete the prediction task. Lv et al. (2015)constructed two homogeneous networks of miRNAs and small molecular drugs. Multiple similarity measurements (i.e., side effect, functional consistency, indication phenotype, and chemical structure) are fused to represent the node feature of miRNAs and drugs, and they implemented the improved random walk restart algorithm on the heterogeneous network, which is fused by two homogeneous networks. Thus, this method can infer the potential MDI without having to resort to the information of known MDI. But there are too many parameters required to adjust in this method. Recently, Deepthi and Jereesh, (2021) developed an ensemble approach of the convolutional neural network based on deep architecture-based classification for identifying the association between miRNAs and drugs. They treated the similarities of miRNAs and drug compounds as the biological features and reduced the dimensions of features by the PCA algorithm. Then, they constructed a convolutional deep neural network for the purpose of feature extraction. Finally, they employed SVM to predict the potential MDIs. Anyway, the aforementioned methods rely heavily on side information calculated by functional similarities such as gene functional similarity and disease phenotype similarity. Abuse of functional similarity carries the risk of label leakage. However, due to the incomplete database, a lot of side information about miRNA and drugs is missing. In most cases, researchers only have the sequence profile and phenotypic profile of biological molecules and chemical compound. Therefore, we think that an MDI prediction method based on the sequence profile rather than functional similarity should be designed.

The information on how miRNAs affect drug effects in the literature can also provide rich information (Fleuren and Alkema, 2015). Hence, Xie et al. (2017) proposed a novel text mining approach named EmDL to infer the MDIs by extracting the explicit information in the literature. They began by splitting substantial articles, which were collected from PubMed and MEDLINE, into individual sentences. For each miRNA–drug pair, the word distance between miRNA and drug appearing in the sentence was calculated to extract the representation features. Last, they leveraged the principal component analysis (PCA) algorithm to reduce the dimension of representation features and was carried out using the support vector machine (SVM) to predict whether the miRNA–drug pair was interactive (Deepthi and Jereesh, 2021). Moreover, Guo et al. (2020) creatively introduced natural language processing (NLP) to the field of biological information. For the purpose of mining the information from the chemical structure of biological entities, they regarded the miRNA sequences and drug SMILES sequences as sentences and implemented the word2vec algorithm for them. However, implementation of NLP methods required a large corpus, and the performance of text mining-based methods will be affected by different corpora and different semantic statements.

**FIGURE 1:**
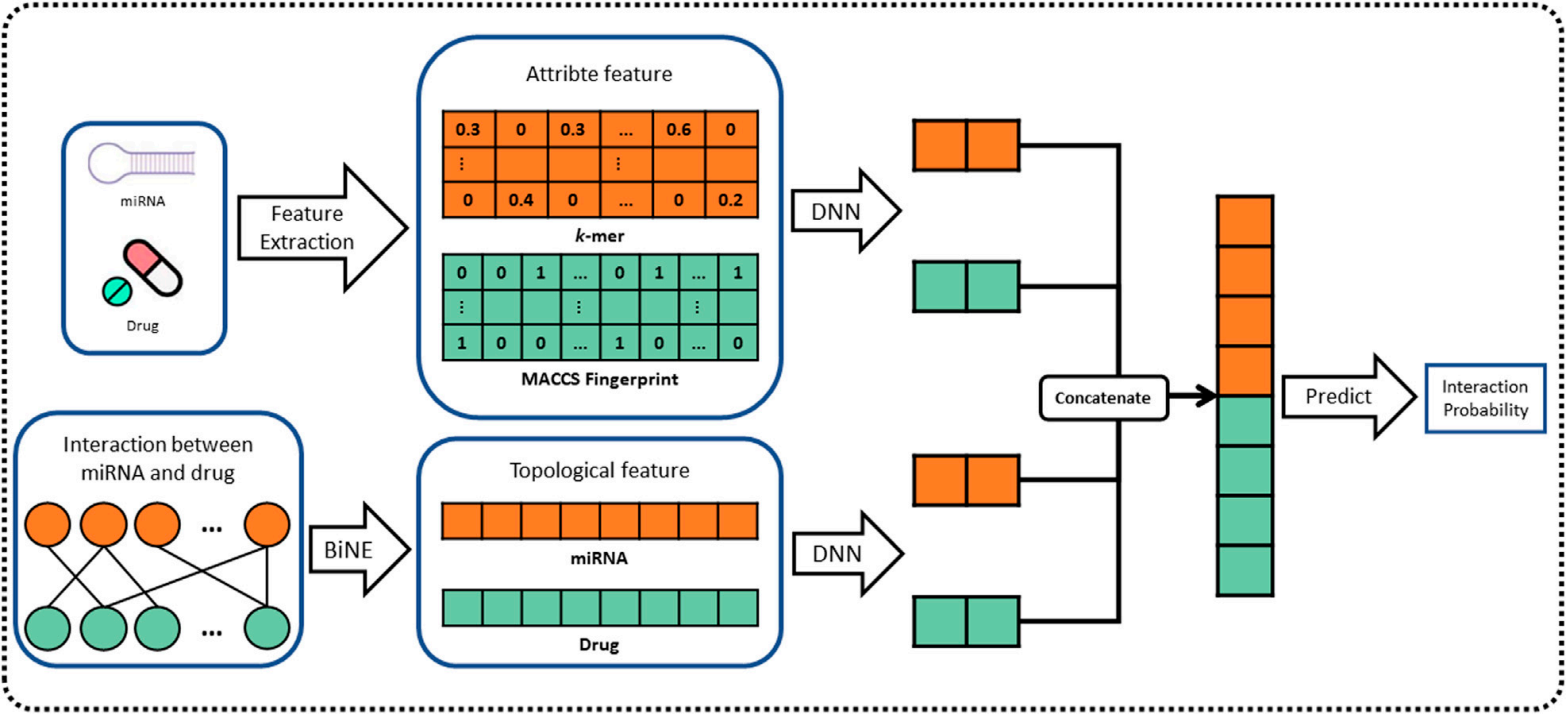
Flowchart of the BNEMDI model for predicting potential MDIs.

In this work, we propose a novel computational method, named BNEMDI, which predicts miRNA–drug interactions using drug substructure fingerprint, miRNA sequence, and MDIs bipartite graph. We have collected known MDI from three databases (e.g., ncDR, RNAInter, and SM2miR) and split them into five datasets. In datasets, the MDI pairs were treated as positive samples, and the same number of unconfirmed miRNA–drug pairs was selected randomly as negative samples. The known MDIs in datasets were constructed as the bipartite graph, and the miRNAs and drug compounds are regarded as the nodes of the graph. The graph embedding methods are pervasive to reveal the complex traits of each entity (Li et al., 2021; Yue and He, 2021). Thus, a graph embedding technique called BiNE was implemented on the bipartite graph for learning the topological features of nodes (Gao et al., 2018), and BNEMDI considers not only the topological information of MDI but also the inherent attribute information of the biological entities. Specifically, the attribute features of drug compounds are denoted by MACCS substructure fingerprints, and the attribute features of miRNAs are calculated by k-mers (Kurtz et al., 2008; Cereto-Massagué et al., 2015). Finally, we constructed a neural network model based on DNN to fuse two kinds of features mentioned earlier and infer the potential miRNA–drug interaction pairs. The flowchart of BNEMDI is shown in Figure 1.

## Materials and Methods

### Dataset

There are several databases about MDIs, for example, the RNA interaction dataset (RNAInter) (Kang et al., 2021), the database for non-coding RNAs involved in drug resistance (ncDR) (Dai et al., 2017), and the database of validated small molecules’ effects on miRNA expression (SM2miR) (Liu X. et al., 2013).

We downloaded a total of 8,053 different experimentally verified miRNA–drug interactions from the three databases mentioned earlier. One thing is to note that the SM2miR database was created on 10 June 2012 and upgraded twice on 28 August 2013 and 27 April 2015. Thus, the SM2miR database was divided into three sub-datasets, according to three versions, named SM2miR1, SM2miR2, and SM2miR3 for convenience, respectively. Therefore, we obtained a total of five datasets and pre-processed them, such as de-redundancy and de-duplication. The details of the three databases are shown in Table 1. We only collected the miRNA–drug interaction pairs of *Homo sapiens* in three databases. The miRNA sequences and drug SMILES are collected from miRBase (Kozomara et al., 2019) and PubChem (Kim et al., 2021a). The drug SMILES is a specification that explicitly describes the molecular structure in ASCII strings (Weininger and sciences, 1988). The drug SMILES are transformed into MACCS fingerprints by the RDKit library.

**TABLE 1:** Statistics of miRNAs, drugs, and miRNA–drug interactions in five datasets.

Dataset	ncDR	RNAInter	SM2miR1	SM2miR2	SM2miR3
Drug	95	281	86	113	142
miRNA	624	1,009	358	536	645
Interaction	4,457	5,739	1,110	1,697	1,940

### Represent MicroRNA With *k*-mer

**FIGURE 2:**
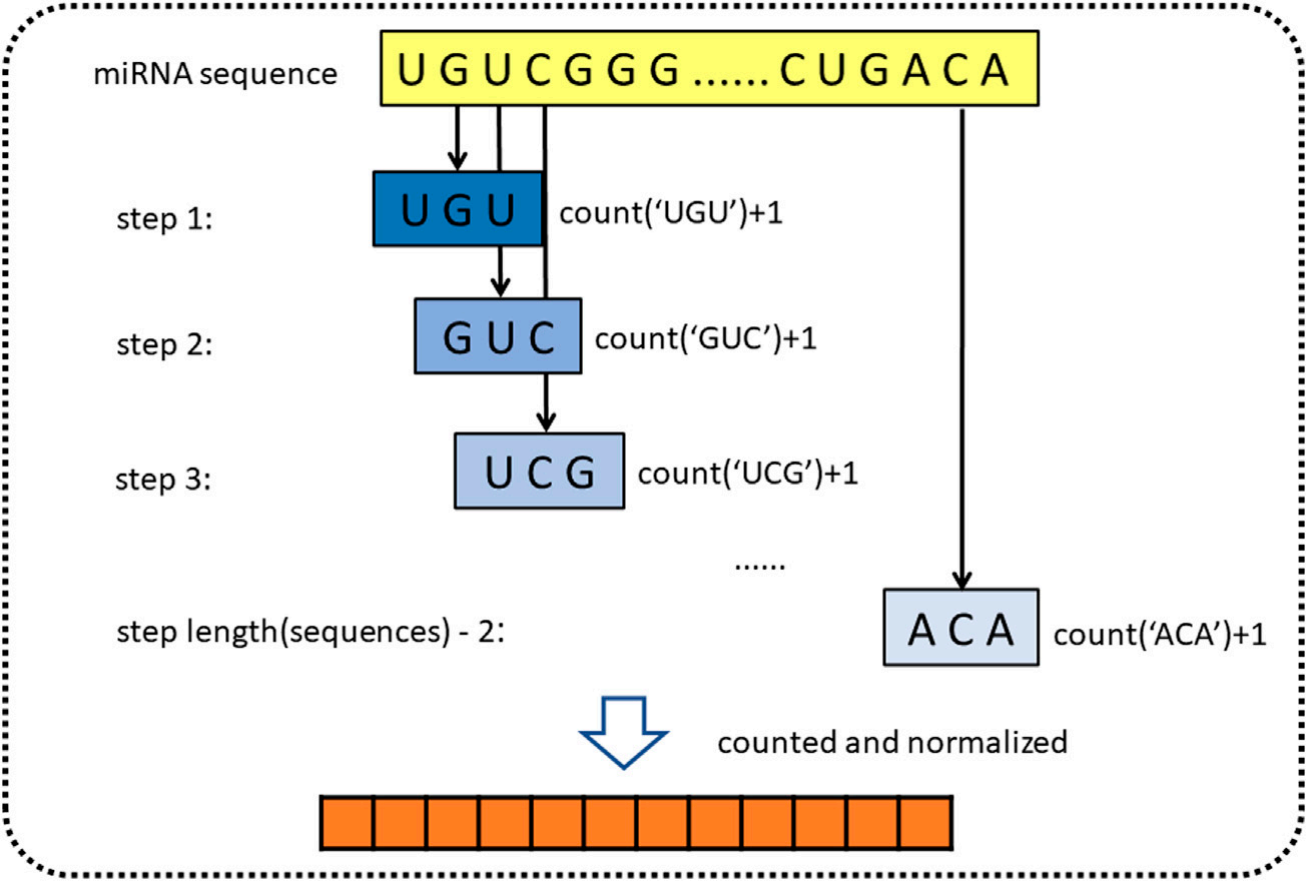
Diagram of *k*-mer for extracting attribute sequences from miRNA sequences.

For obtaining genomic information on miRNA, the sequence of miRNA is represented by *k*-mer (Liu B. et al., 2013). *k*-mer is a feature representation method, which is widely used in the field of bioinformatics. Yousef et al. (2017) used *k*-mer to construct simple sequence-based features to describe miRNAs for miRNA categorization (Yousef et al., 2017; Erten-Ela et al., 2018). In addition, Yi et al. (2020) also used *k*-mer to represent molecules such as lncRNA, miRNA, and protein in the molecule association network (Yi et al., 2020; Pan et al., 2022). *k*-mer is a substring of biological sequence with a length of *k*. For the miRNA sequences, we define the *3*-mer of miRNA as the subsequence, such as “AGG” and “AAA.” Then we sequentially extract three nucleotides from the first nucleotides, using the form of a sliding window (step length is one). Since miRNA consists of four types of bases, there are 64 $\left(4^{3}\right)$ possible *3*-mer patterns in a sequence. After that, we count the normalized frequencies of all *3*-mer patterns. Finally, we obtained miRNA representation vectors with a length of 64 and containing miRNA sequence information. Figure 2 shows the principle of *k*-mer.

### Represent Drug Molecules With MACCS Fingerprint

**FIGURE 3:**
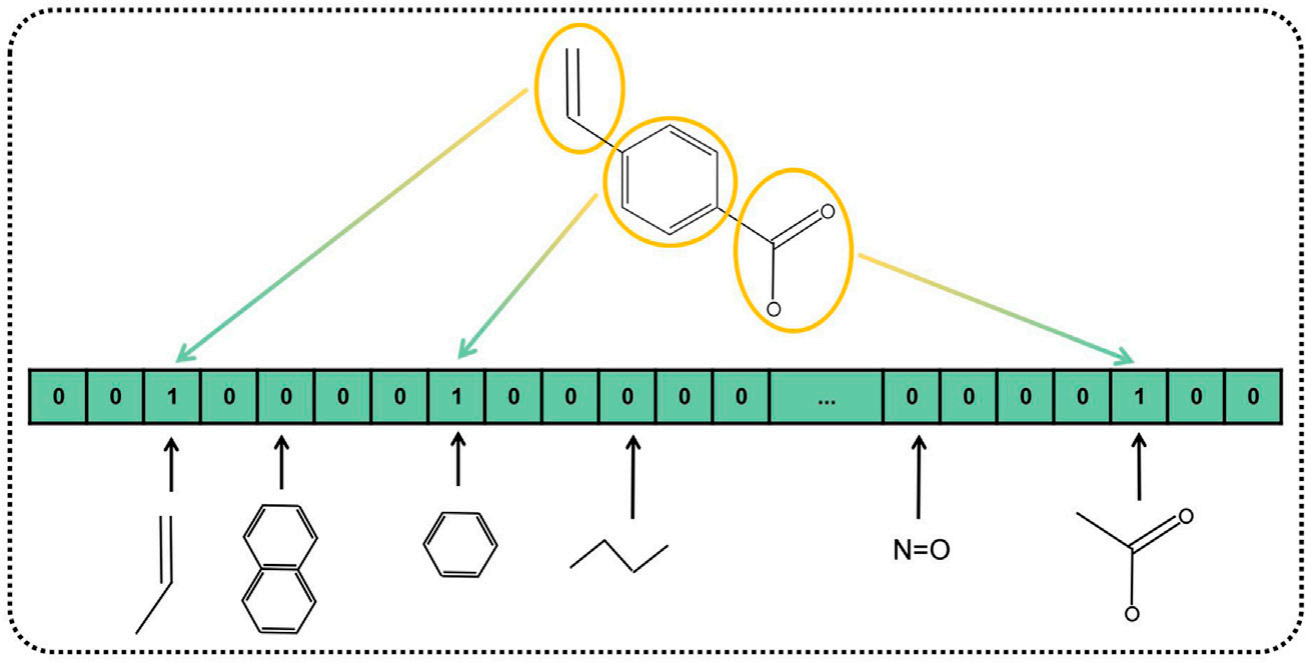
Diagram of MACCS fingerprint-represented drug substructures.

In the past research, numerous kinds of descriptors have been established to portray the chemical structure of pharmaceutical compounds such as geometrical, topological constitutional, and quantum chemical properties (Cao et al., 2012). The substructure keys-based fingerprint is customarily adopted as the descriptor to represent the chemical structure. Substructure fingerprints encode molecular structure to a bit-string with a fixed length, according to the substructure of the drug instead of using 3D structural information. Plenty of previous research works have demonstrated that substructure fingerprint is effective and feasible to represent drugs. Specifically, we incorporated a dictionary that includes a list of substructure features represented as SMART strings. SMART is a system to identify substructures by the expanding rule of SMILES. After the first step of composing the dictionary, we compare each item of the dictionary to the given molecular substructures, if the SMART pattern is included in the given molecular substructure, the corresponding bit of fingerprint is set to one, and zero otherwise. An example of the substructure fingerprint determined by the given molecular substructure is displayed in Figure 3. Herein, we used MACCS fingerprint to compose the dictionary, which contained 166 types of general molecular substructures and covered most of the interesting chemical structures of drugs. Finally, we represented Boolean vectors of molecular drug for the length of 166.

### Topological Features Extraction Based on Graph Representation Method

In this study, the graph representation learning method may encode each node by topological information and embed nodes in a low-dimension space. It is different from previous studies, in which it can extract underlying information from the network.

The challenge of MDI prediction may be formulated as a link prediction problem with a heterogeneous graph. The MDIs network is employed to construct a heterogeneous graph G=(D,M,E) , and there are two types of nodes, drug $\text{D}=\left\{{\text{d}}_{1},d_{2},\ldots ,d_{i}\right\}$ ( i is the index of drugs in the dataset) and miRNA $M=\left\{m_{1},m_{2},\ldots ,m_{j}\right\}$ ( j is the index of miRNA in the dataset). E⊂D×M denotes the set of edges between D and M . The edges represent the known interactions between drugs and miRNAs. If $d_{i}$ and $m_{j}$ have interaction, the weight of the edge is set to one, and zero otherwise. The matrix $W=\left[w_{ij}\right]$ denotes the weight of the edges between drug $d^{i}$ and miRNA $m^{j}$ in graph G. The graph embedding aims to look for a map function $f:D\cup M\to R^{t}$ , where t<<|m|∪|d| . In other words, the low-dimensional presentation vectors of each node in the graph will be learned by the graph embedding method, and maintain the graph topology information and node properties (Cai et al., 2018). To achieve this aim, we utilized a graph embedding method called BiNE, which has great performance in reconstructing the original bipartite network, proposed by Gao et al. (2018). Previous research on graph embedding has raised the question of extracting explicit relations between the nodes of different types and implicit relationships among the nodes of sample types. BiNE contributed an innovative idea to solve this problem by constructing a jointly optimizing framework, consisting of three objection functions and variable weight. These three objective functions include an explicit relation and two implicit relations.

To model the explicit relations, we calculated the local proximity between two different vertices in the bipartite network, which is based on local proximity in LINE (Tang et al., 2015). We define the joint probability between two connected nodes as:

$$P\left(i,j\right)=\frac{w_{ij}}{\sum_{e_{ij}\in E}w_{ij}}$$ (1)

where $w_{ij}$ is the weight of the edge between two types of nodes.

Drawing on the principle of word2vec, BiNE estimates the local proximity between two nodes by inner product (Church, 2017), and the sigmoid function is used to map the interaction value to the probability space. The joint probability of two different types of nodes in embedding space is defined as follows:

$$\overset{\wedge }{P}\left(i,j\right)=\frac{1}{1+\exp\left(-d_{i}^{T}m_{j}\right)},$$ (2)

where ${\vec{d}}_{i}\in R^{t}$ and ${\vec{m}}_{j}\in R^{t}$ are the embedding vectors of drugs $d_{i}$ and miRNAs $m_{j}$ , respectively.

To get the knowledge of observed edges and learn the embedding vectors, we need to minimize the difference between empirical distribution and the reconstructed distribution. KL-divergence is used to measure the difference between the previously two joint probabilities mentioned. The first part of the joint optimizing framework can be defined as:

$$minimizeO_{1}=KL\left(P||\overset{⌢}{P}\right)=\sum_{e_{ij}\in E}P\left(i,j\right)\log\left(\frac{P\left(i,j\right)}{\overset{⌢}{P}\left(i,j\right)}\right).$$ (3)

Studies of recommendation systems demonstrated that implicit relations are also helpful to discover potential information in the heterogeneous graph as explicit relations (Jiang et al., 2016; Yu et al., 2018). This means that nodes of the same type are not connected in the bipartite network, but still contain a wealth of information, that is, crucial to model the implicit relationship between the nodes of the same type. BiNE constructs two homogeneous networks in accordance with the interaction profile between two types of nodes and performs the random walk on two homogeneous networks to encode the high-order proximity of the origin network.

To reveal the 2nd proximity of the heterogeneous graph, BiNE utilizes co-HITS (Deng et al., 2009) to generate two weighted homogeneous networks (drug–drug network and miRNA–miRNA network). In accordance with co-HITS, the correlation coefficient between two nodes can be defined as:

$$w_{ij}^{M}=\sum_{k\in D}w_{ik}w_{jk};w_{ij}^{D}=\sum_{k\in M}w_{ki}w_{kj}$$ (4)

where $w_{ij}$ is the weight of the edge $e_{ij}$ . Intuitively, suppose an i×j MDI bipartite matrix $G_{b}$ , the drug–drug network can be denoted by a i×i matrix $G_{b}G_{b}^{T}$ , and the miRNA–miRNA network can be represented by a j×j matrix $G_{b}^{T}G_{b}$ .

Truncated random walks are employed on two homogeneous networks previously generated to obtain the corpus of node sequences. Therefore, the biased and self-adaptive random walk generator, which may maintain the vertex distribution, is introduced to produce the corpus of node sequences with true validity and effectiveness. Its core design can be described as “richer get richer.” Specifically, the greater centrality of a node, the more likely that node will be the starting point for the random walk to begin. The centrality of nodes in the homogeneous network is measured by HITS (Kleinberg, 1999). Compared with other random walk-based measures, a probability is specified to stop the random walk in each step. Therefore, the node sequence generated by our method does not have a fixed length because the variable-length sequences are more simulated to natural language.

The skip-gram model is carried out to process the samples of two corpora obtained from truncated random walk. If two nodes frequently appear in the same context of a node sequence, the skip-gram model will assign them similar embedding vectors.

In order to learn the implicit relations, two objection functions are defined to maintain the high-order proximity by maximizing the conditional probability. The symbols of $C_{S}\left(d_{i}\right)$ and $C_{S}\left(m_{j}\right)$ represent the context of node $d_{i}$ and $m_{j}$ in a sequence S , respectively. For the corpus of the drug homogeneous network $P^{D}$ , the objection function is as follows:

$$maximizeO_{2}=\prod_{d_{i}\in S\wedge P^{D}}\prod_{d_{c}\in C_{S}\left(d_{i}\right)}P\left(d_{c}|d_{i}\right)$$ (5)

Then, the corpus of the miRNA homogeneous network $P^{M}$ is treated in the same way, and the objection function is expressed as:

$$maximizeO_{3}=\prod_{m_{j}\in S\wedge P^{M}}\prod_{m_{c}\in C_{S}\left(m_{j}\right)}P\left(m_{c}|m_{i}\right)$$ (6)

Similar to the LINE (Tang et al., 2015), the conditional probability $P\left(d_{c}|d_{i}\right)$ and $P\left(m_{c}|m_{i}\right)$ are defined using the inter product kernel and softmax function:

$$P\left(d_{c}|d_{i}\right)=\frac{\exp\left({\vec{d}}_{i}^{T}{\vec{\theta }}_{c}\right)}{\sum_{k=1}^{\left|D\right|}\left({\vec{d}}_{i}^{T}{\vec{\theta }}_{k}\right)},P\left(m_{c}|m_{i}\right)=\frac{\exp\left({\vec{m}}_{j}^{T}{\vec{\vartheta }}_{c}\right)}{\sum_{k=1}^{\left|M\right|}\left({\vec{m}}_{j}^{T}{\vec{\vartheta }}_{k}\right)}$$ (7)

where |D| and |M| represent the number of drug compounds and miRNAs, respectively. The context vectors corresponding to two types of nodes are denoted as ${\vec{\theta }}_{c}$ and ${\vec{\vartheta }}_{c}$ .

Finally, the three components of the objective function are combined into the joint optimization framework for learning the low-dimension embedding vectors of the bipartite network. The overall jointly optimizing function is defined as follows:

$$maximizeL=\alpha \log O_{2}+\beta \log O_{3}-\gamma O_{1}$$ (8)

where α , β , and γ are parameters of explicit relation and implicit relation.

To improve computational efficiency, a negative sampling method is adopted to approach the complicated denominator of the sigmoid function. In particular, nodes are divided into different buckets by locality-sensitive hashing (LSH) (Wang et al., 2013) and randomly selected as the negative samples. Finally, the joint framework is optimized by the stochastic gradient ascent (SGA) algorithm. The first part of the optimizing framework $L_{1}=-\gamma O_{1}$ is maximized to update embedding vectors ${\vec{d}}_{i}$ and ${\vec{m}}_{j}$ , and the updated rules of embedding vectors ${\vec{d}}_{i}$ and ${\vec{m}}_{j}$ are expressed as follows:

$${\vec{d}}_{i}={\vec{d}}_{i}+\lambda \left\{\gamma w_{ij}\left[1-\sigma \left({\vec{d}}_{i}^{T}{\vec{m}}_{j}\right)\right]\cdot {\vec{m}}_{j}\right\}$$ (9)

$${\vec{m}}_{j}={\vec{m}}_{j}+\lambda \left\{\gamma w_{ij}\left[1-\sigma \left({\vec{d}}_{i}^{T}{\vec{m}}_{j}\right)\right]\cdot {\vec{d}}_{i}\right\}$$ (10)

where λ represents the learning rate, and σ represents the sigmoid function. Then, the part of $\alpha \log O_{2}$ and $\beta \log O_{3}$ are also maximized to update embedding vectors ${\vec{d}}_{i}$ and ${\vec{m}}_{j}$ to follow the rules:

$${\vec{d}}_{i}={\vec{d}}_{i}+\lambda \{\sum_{z\in \{d_{c}\}\cup N_{S}^{ns}\left(d_{i}\right)}\alpha [I\left(z,d_{i}\right)-\sigma \left({\vec{d}}_{i}^{T}{\vec{\theta }}_{z}\right)]•{\vec{\theta }}_{z}\},$$ (11)

$${\vec{m}}_{j}={\vec{m}}_{j}+\lambda \{\sum_{z\in \{m_{c}\}\cup N_{S}^{ns}\left(m_{j}\right)}\beta [I\left(z,m_{j}\right)-\sigma \left({\vec{m}}_{j}^{T}{\vec{\vartheta }}_{z}\right)]•{\vec{\vartheta }}_{z}\},$$ (12)

where $I\left(z,d_{i}\right)$ and $I\left(z,m_{j}\right)$ is an indicator function that confirms whether the node z belongs to the context of $d_{i}$ and $m_{j}$ , respectively. The context of nodes is updated as:

$$\vec{\theta }z=\vec{\theta }z+\lambda \left\{\alpha \left[I\left(z,d_{i}\right)-\sigma \left({\vec{d}}_{i}^{T}{\vec{\theta }}_{z}\right)\right]•{\vec{d}}_{i}\right\}$$ (13)

$$\vec{\vartheta }z=\vec{\vartheta }z+\lambda \left\{\beta \left[I\left(z,m_{j}\right)-\sigma \left({\vec{m}}_{j}^{T}{\vec{\vartheta }}_{z}\right)\right]•{\vec{m}}_{j}\right\}$$ (14)

### Building Predictor

In this section, we will introduce how to predict whether the miRNA–drug pairs have underlying interaction. After feature extraction, the attribute and topological features of miRNAs and drugs were concatenated and fed into the DNN model for fusing as unified dimension representation vectors. Finally, a dense layer with 256 neurons is used to complete the classification task. Specifically, suppose that the nodes miRNA and nodes drug are $d_{i}$ and $m_{j}$ , and the representation features of them are $f_{i}$ and $f_{j}$ , respectively. The possibility of interaction between $d_{i}$ and $m_{j}$ can be defined as:

$$P^{ij}=\sigma \left(f_{i}^{T}⊕f_{j}\right)$$ (15)

where σ means the sigmoid function and ⊕ means the concatenation. $P^{ij}$ represent the prediction score between $d_{i}$ and $m_{j}$ , if the $P^{ij}$ is greater than 0.5 means, $d_{i}$ is to interact with $m_{j}$ , and vice versa. The binary cross-entropy was used as the loss function, and the “Adam” algorithm was used to optimize the model.

## Results and Discussion

### Evaluation Criteria

As MDI prediction is a binary classification problem for each pair of miRNA and drugs, we used some evaluation criteria to measure the performance of the proposed model, including accuracy (Acc.), sensitivity (Sen), specificity (Spec.), also precision (Prec.), and Matthews correlation coefficient (MCC). They are defined as:

$$Acc.=\frac{TN+TP}{TN+TP+FN+FP}$$ (16)

$$Sen.=\frac{TP}{FP+FN},$$ (17)

$$Spec.=\frac{TN}{TN+FP},$$ (18)

$$Prec.=\frac{TP}{TP+FP}$$ (19)

$$MCC=\frac{TP\times TN-FP\times FN}{\sqrt{\left(TP+FP\right)\left(TP+FN\right)\left(TN+FP\right)\left(TN+FN\right)}}$$ (20)

Here, TP and TN are signs of the number of correct positive samples and correct negative samples predicted by the model, respectively. Correspondingly, FP and FN are signs of the number of false positive samples and false negative samples predicted by the model, respectively (Pan et al., 2020). Following previous studies, the receiver-operating characteristic (ROC) and precision-recall (PR) are implemented to visually display the result of the experiment, and the area under ROC (AUC) and PR (AUPR) are used to assess the comprehensive performance of the proposed model.

### Prediction Performance on Different Datasets

**FIGURE 4:**
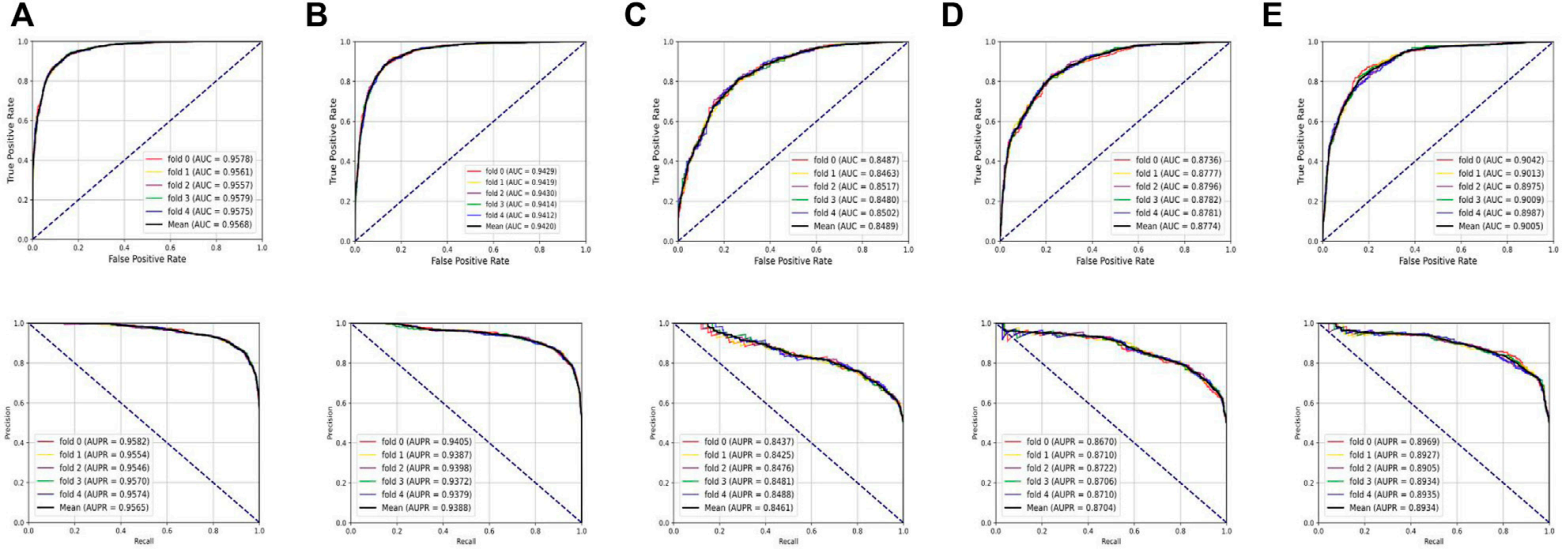
Prediction performance of BNEMDI based on ROC and PR curves. **(A)** Five-fold cross-validation ROC and PR curves on the ncDR dataset. **(B)** Five-fold cross-validation ROC and PR curves on the RNAInter dataset. **(C)** Five-fold cross-validation ROC and PR curves on the SM2miR1 dataset. **(D)** Five-fold cross-validation ROC and PR curves on the SM2miR2 dataset. **(E)** Five-fold cross-validation ROC and PR curves on the SM2miR3 dataset.

To systematically evaluate the performance of the BNEMDI model, our proposed model is implemented to predict potential MDI pairs on five different datasets, and five-fold cross-validations are implemented for obtaining a more accurate assessment. In detail, the dataset will be divided into five parts, each part will serve as the testing set in turn, and the rest as training sets. Afterward, Table 2 lists various evaluation values to illustrate the prediction performance of BNEMDI. As can be seen in Table 2, we get the result of the experiment with the accuracy of 88.75% (ncDR), 87.23% (RNAInter), 77.24% (SM2miR1), 79.92% (SM2miR2), and 81.86% (SM2miR3). The standard deviations of accuracy are 0.1, 0.34, 0.39, 0.21, and 0.65%, respectively. To directly illustrate the prediction performance of BNEMDI on each dataset, Figure 4 presents the ROC and PR curves of the result of five-fold cross-validations on five datasets. The proposed model BNEMDI achieves average AUCs of 0.9568 (ncDR), 0.9420 (RNAInter), 0.8489 (SM2miR1), 0.8774 (SM2miR2), and 0.9005 (SM2miR3). The standard deviations of five-fold cross-validations are 0.001, 0.0016, 0.0021, 0.0023, and 0.0026, respectively. It is apparent from these criteria values that our proposed model BNEMDI is stable and effective.

**TABLE 2:** Performance of the proposed method on five datasets.

Fold	AUC	AUPR (%)	Acc (%)	Sen (%)	Spec (%)	Prec (%)	MCC (%)
ncDR	0.9568 ± 0.0010	95.65 ± 0.13	88.75 ± 0.10	89.13 ± 0.19	88.39 ± 0.13	88.47 ± 0.11	77.51 ± 0.20
RNAInter	0.9420 ± 0.0016	93.88 ± 0.16	87.23 ± 0.34	88.99 ± 1.05	85.47 ± 1.30	85.98 ± 0.94	74.52 ± 0.65
SM2miR1	0.8489 ± 0.0021	84.61 ± 0.25	77.24 ± 0.39	80.82 ± 0.33	73.66 ± 0.68	75.42 ± 0.49	54.62 ± 0.78
SM2miR2	0.8774 ± 0.0023	87.04 ± 0.17	79.92 ± 0.21	81.12 ± 0.33	78.73 ± 0.23	79.22 ± 0.19	59.86 ± 0.42
SM2miR3	0.9005 ± 0.0026	89.34 ± 0.20	81.86 ± 0.65	79.47 ± 1.42	84.24 ± 2.05	83.49 ± 1.62	63.81 ± 1.37

Previously, some studies have conducted MDI prediction experiments on the ncDR dataset (Huang et al., 2018; Huang et al., 2020). Herein, we compared our proposed model with these models and some classical methods like collaborative filtering (CF) and matrix factorization (MF) (Boutsidis and Gallopoulos, 2008; Su and Khoshgoftaar, 2009). The evaluation criteria are AUC and the results are shown in Table 3. In GCMDR and HMDPI, the attribute features of miRNAs and drugs were constructed using miRNA expression profile, drug substructure fingerprints, gene ontology, and disease ontology. Huang et al*.* constructed the GCMDR model by combining graph convolution and auto-encoder to learn deep features. In the GCMDR model, the dimensional latent factor, units in hidden layer, maximum Chebyshev polynomial degree, and training epochs are set to 25, 100, 3, and 200, respectively. In EPLMI, they implemented a two-way diffusion method on the weighted network to generate resource vectors which can be defined as:

$$R_{\ln cRNA}=\sum_{m=1}^{nm}\frac{A_{a,m}^{w}\cdot A_{*,m}}{\sum_{i=1}^{nl}A_{i,m}^{w}}$$ (21)

$$R_{miRNA}=\sum_{l=1}^{nl}\frac{A_{l,b}^{w}\cdot A_{l,*}}{\sum_{i=1}^{nm}A_{l,i}^{w}}$$ (22)

where $A^{w}$ is the weighted adjacency matrixes constructed by similarity, and A is the adjacency matrixes. Other experimental parameters are set to default.

**TABLE 3:** Comparison of the prediction performance based on the ncDR dataset (N/A means not available).

Method	ncDR	RNAInter	SM2miR1	SM2miR2	SM2miR3
GCMDR	0.9359 ± 0.0006	N/A	N/A	N/A	N/A
EPLMI	0.8971 ± 0.0009	N/A	N/A	N/A	N/A
Neighbor-based CF	0.8644 ± 0.0009	0.8532 ± 0.0007	0.6289 ± 0.0017	0.7346 ± 0.0027	0.8654 ± 0.0015
Drug-based CF	0.7313 ± 0.0008	0.7120 ± 0.0010	0.6982 ± 0.0026	0.6993 ± 0.0013	0.7030 ± 0.0016
miRNA-based CF	0.8235 ± 0.0015	0.8364 ± 0.0022	0.6325 ± 0.0019	0.6534 ± 0.0014	0.7644 ± 0.0009
SVD-based MF	0.6007 ± 0.0052	0.6189 ± 0.0044	0.5978 ± 0.0050	0.6039 ± 0.0051	0.6045 ± 0.0045
BNEMDI	0.9568 ± 0.0010	0.9420 ± 0.0016	0.8489 ± 0.0021	0.8774 ± 0.0023	0.9005 ± 0.0026

In the methods based on CF, the self-similarities of miRNA and drug are calculated by the Pearson correlation coefficient (PCC), which is defined as:

$$P_{*}\left(a,b\right)=\frac{\sum_{i=1}^{N}\left(f_{ai}-\bar{f_{a}}\right)\left(f_{bi}-\bar{f_{b}}\right)}{\sqrt{\sum_{i=1}^{N}{\left(f_{ai}-\bar{f_{a}}\right)}^{2}{\sum_{i=1}^{N}\left(f_{bi}-\bar{f_{b}}\right)}^{2}}},$$ (23)

where $f_{a}$ and $f_{b}$ represent the features of two same types of elements (miRNA or drug). Based on the PCC, self-similarity matrixes and adjacency matrixes M for miRNA and drug, the predicted score matrix of drug-based CF can be defined as:

$$M_{drug}^{\prime }\left(d_{i},m_{j}\right)=\frac{\sum_{k=1}^{n_{d}}P_{drug}\left(d_{i},d_{k}\right)\cdot M_{k,j}}{n_{d}}$$ (24)

where M′ is the predicted matrix and $n_{d}$ is the number of drugs in the dataset.

Correspondingly, the predicted score matrix of miRNA-based CF can be defined as:

$$M_{miRNA}^{\prime }\left(d_{i},m_{j}\right)=\frac{\sum_{k=1}^{n_{m}}P_{miRNA}\left(m_{i},m_{k}\right)\cdot M_{i,k}}{n_{m}}s$$ (25)

Neighbor-based CF takes into account both drug-based CF and miRNA-based CF and is defined as:

$$M_{neighbor}^{\prime }\left(d_{i},m_{j}\right)=\frac{M_{drug}^{\prime }\left(d_{i},m_{j}\right)+M_{miRNA}^{\prime }\left(d_{i},m_{j}\right)}{2}$$ (26)

Several studies on drug target interaction prediction or drug repositioning have used similarity-related information to construct the prediction models. Although they gain optimistic results on datasets, it seems difficult for the model to work in real-world scenarios. However, the similarity itself is related to interactions of biological entities, and the abuse of similarity will potentially lead to label leakage. The prediction ability of label leaking models is easily overestimated when it implements on a known dataset. In this experiment, after dividing the dataset into training sets and test sets, only the training set was extracted topological features and used to construct the prediction model for avoiding label leakage. For instance, there are 4,457 MDI pairs in the ncDR dataset, of which only 3,565 MDI pairs will be extracted as features and used to construct the prediction model. But there are no such issues in the attribute features.

### Ablation Experiment

To better construct representation vectors, we considered attribute features and topological features of nodes in the miRNA–drug bipartite network. In this section, we are going to discuss the impact of different features on the performance of BNEMDI. We consider three kinds of features: attribute feature, topological feature, and the combination of them to separately construct the representation vectors and the corresponding prediction model. The accuracy is used as the standard to compare the influence of various features on the model.

**FIGURE 5:**
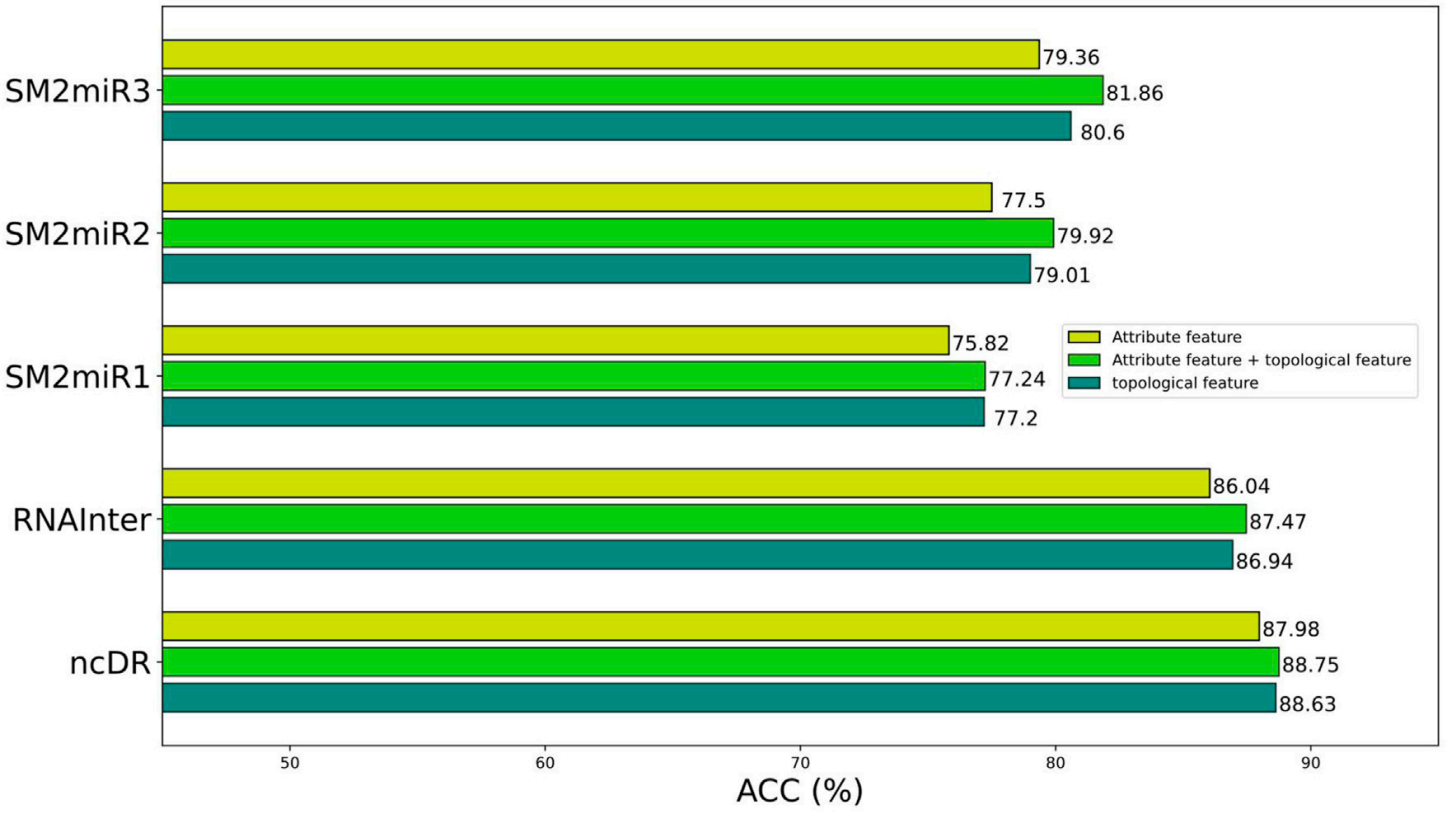
Prediction performance of different features on different datasets.

Figure 5 shows the prediction performance of models based on different features. In general, the topological features are more effective than the attribute features. Therefore, we concluded that the topological features make a great contribution to the proposed model. Although attribute features do not perform as well as the topological feature, the production of attribute features only requires sequence information like SMILES and miRNA sequences. Thus, the attribute features are suitable as the representation vectors for the new samples.

The attribute features are constructed by the sequence profile information of nodes in the relationship network and contain chemical structure information of the miRNAs and drugs. The topological features consider high-order implicit transition relationships and explicit relations, which provide distinct similarity information of homologous nodes. This makes it easier for miRNA and drug relationship pairs with similar structures to known MDI to be considered interacting, and vice versa. In principle, the combination of topological features and attribute features will make the effect more pronounced.

### Compare With Other Embedding Methods and Classifiers

The topological feature extracted by BiNE is important for building the BNEMDI model. To highlight the advantages of BiNE, we compare BiNE to three state-of-the-art graph representation methods and discuss their performance in different dimensions. In a similar way to BiNE, several state-of-the-art network embedding methods (i.e., DeepWalk (Perozzi et al., 2014), LINE (Tang et al., 2015), and node2vec (Grover and Leskovec, 2016)) are used to learn the embedding vectors of each node and compare to BiNE. DeepWalk carries out the random walk on the graph to generate node sequences, and the node sequences are regarded as sentences to learn embedding vectors by word2vec (Church, 2017). LINE combines the first-order and second-order proximities and optimizes them using the asynchronous stochastic gradient algorithm (ASGD) (Recht et al., 2011). Node2vec is an extension of DeepWalk. It introduces depth-first search (DFS) and breadth-first search (DFS) to the process of the random walk. BFS may explore the structural properties of the graph, and DFS may reflect the homogeneity between similar nodes. Based on the experiment, the best result may be obtained when hyper-parameters p and q are set to 0.5 (Gao et al., 2018). Moreover, the parameters of other embedding methods are set to their default settings except for the dimension of the node embedding vector.

**FIGURE 6:**
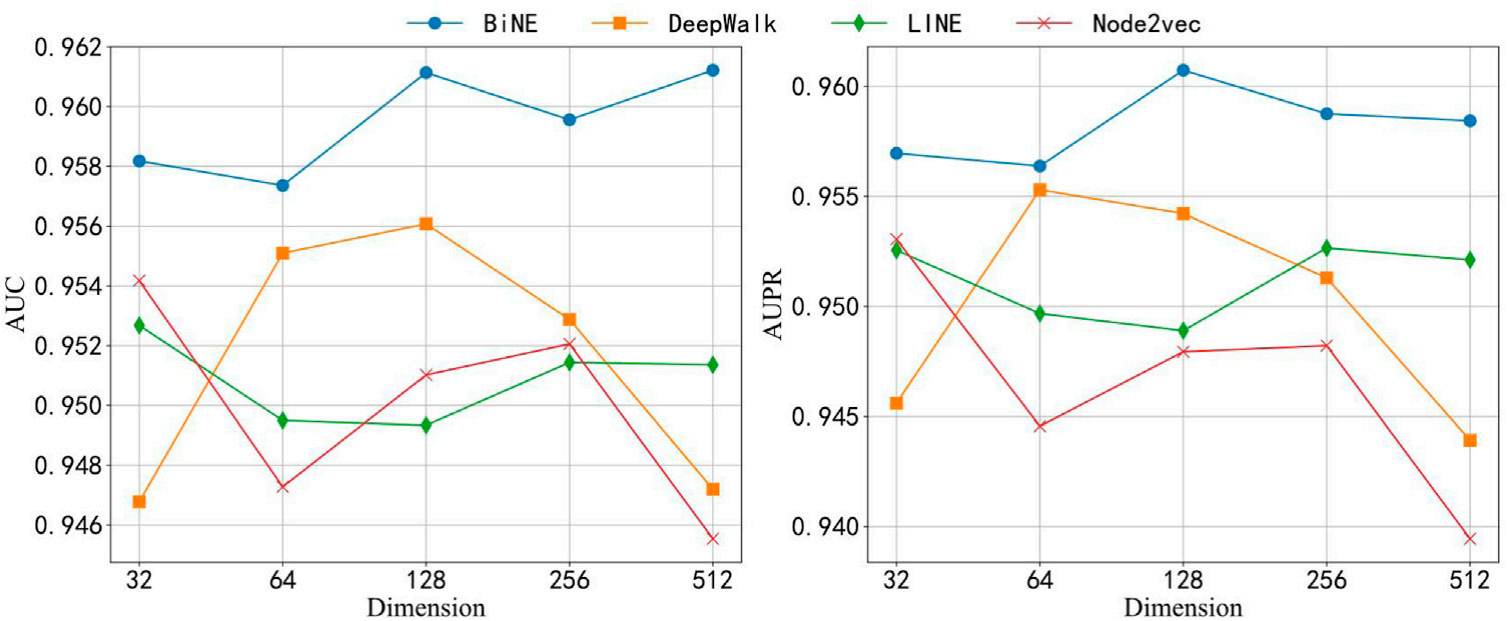
AUC and AUPR of four network embedding methods in different dimensions.

Here, we analyze the performance of models in different dimensions of the node embedding vector. We have carried on the experiment to each embedding method separately in five different dimensions, 32, 64, 128, 256, and 512. We also employed these embedding approaches to learn the topological features from the bipartite network and combine the attribute features of drug compounds and miRNAs to construct this prediction model. Figure 6 shows the results of each model that was applied to the ncDR dataset. The y axis of Figure 6 depicts the AUC and AUPR of each prediction model, and the x-axis depicts five kinds of node-embedding dimensions. According to Figure 6, we can draw a conclusion that the model with the BiNE embedding method gets the best result among these methods. The main reason for the outstanding performance of BiNE is that it considers unique information of drug and miRNA nodes while processing the relations in the miRNA–drug bipartite network. BiNE calculates the second-order proximity of nodes in the miRNA–drug bipartite network to learn the implicit relation between drugs and miRNAs, which can get more efficient similarity compared with the similarities based on domain knowledge (Yue and He, 2021).

When the dimension of embedding vectors is 64, the BiNE model achieves the lowest AUC and PR values of 0.957 and 0.956, respectively. To avoid overfitting, the dimension of embedding vectors generated by the BiNE model was set to 64 in the subsequent experiments.

We further evaluate the impact of the classifier on the overall model by comparing it with several popular machine learning classifiers, including random forest (RF), naive Bayes (NB), logistics regress (LR), and SVM classifiers (Gui et al., 2015). The features extracted by the same method of the proposed model were used as the input of the aforementioned classifiers for five-fold cross-validations on the ncDR dataset.

**FIGURE 7:**
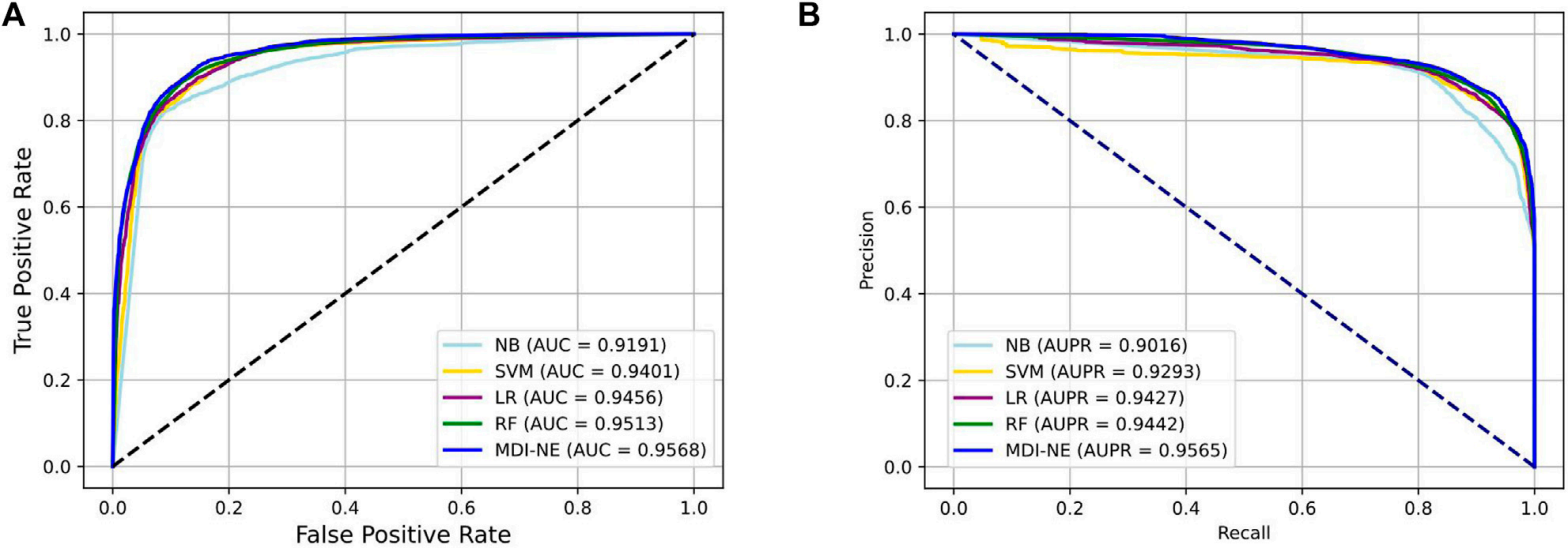
Comparison of BNEMDI with different classifiers under five-fold cross-validation. **(A)** ROC curve on the ncDR MDI dataset. **(B)** PR curve on the ncDR MDI dataset.

Table 4 exhibits the average performance of the five-fold cross-validations of each classifier on the ncDR dataset. As shown in Table 4, NB, SVM, LR, and RF obtained an average accuracy of 86.49, 86.84, 87.56, and 88.38%, respectively. The BNEMDI achieved the highest accuracy of 88.75%. We gained an average AUC score of 0.9167, 0.9416, 0.9473, 0.9505, and 0.9573, and an average PR score of 94.27, 90.16, 94.40, 92.93, and 95.65% for NB, LR, RF, and BNEMDI. For a more intuitive comparison, Figure 7 depicts the corresponding ROC and PR curves. The proposed model leads in most evaluation metrics with the highest AUC of 0.9573 and the highest AURR of 0.9565 and has a relatively low standard deviation. Synthetically, BNEMDI not only has an excellent performance in various evaluation criteria but also is more stable than other classifiers.

**TABLE 4:** Average performance of the different classifiers on ncDR datasets.

Classifier	AUC	AUPR (%)	Acc (%)	Sen (%)	Spec (%)	Prec (%)	MCC (%)
NB	0.9166 ± 0.0035	90.16 ± 0.53	86.49 ± 0.54	81.69 ± 1.22	91.30 ± 0.87	90.38 ± 0.81	73.34 ± 1.05
SVM	0.9415 ± 0.0033	92.93 ± 0.57	86.84 ± 0.63	85.91 ± 0.38	87.77 ± 1.21	87.55 ± 1.08	73.70 ± 1.28
LR	0.9473 ± 0.0029	94.27 ± 0.48	87.56 ± 0.77	86.56 ± 0.32	88.56 ± 1.45	88.34 ± 1.32	75.14 ± 1.56
RF	0.9502 ± 0.0036	94.42 ± 0.56	88.38 ± 0.87	88.18 ± 0.88	88.58 ± 1.79	88.56 ± 1.58	76.77 ± 1.76
BNEMDI	0.9573 ± 0.0009	95.65 ± 0.13	88.75 ± 0.10	89.13 ± 0.19	88.39 ± 0.13	88.47 ± 1.11	77.51 ± 0.20

## Case Study

In this subsection, we carried out a case study on the RNAInter dataset. All of the known MDIs were used to construct representation vectors to predict all candidate miRNA–drug pairs in the dataset. Then, we ranked these candidate miRNA–drug pairs according to the predicted scores in the descending order. The top 30 predicted relationships are shown in Table 5. Among the top 10, 20, and 30 predicted relationships, 7, 12, and 18 relationships are verified by the previous literature in PubMed, respectively.

**TABLE 5:** Top 30 potential MDIs predicted by BNEMDI.

Drug (CID)	miRNA	Evidence	Drug (CID)	miRNA	Evidence
60750	hsa-miR-24-3p	Unconfirmed	60750	hsa-miR-29c-3p	29807360
60750	hsa-miR-205-5p	31602229	2767	hsa-miR-1236-3p	30805558
2767	hsa-miR-193b-3p	27918099	5310940	hsa-miR-660-5p	Unconfirmed
3385	hsa-miR-10a-5p	Unconfirmed	60750	hsa-miR-532-5p	Unconfirmed
3385	hsa-miR-33b-5p	Unconfirmed	31703	hsa-miR-18a-5p	Unconfirmed
3385	hsa-miR-376a-3p	Unconfirmed	3385	hsa-miR-431-5p	Unconfirmed
2520	hsa-miR-126-3p	Unconfirmed	5310940	hsa-miR-196a-5p	Unconfirmed
60750	hsa-miR-124-3p	35127724	5310940	hsa-miR-101-3p	31934027
3385	hsa-miR-93-5p	30573973	60750	hsa-miR-1908-5p	Unconfirmed
31703	hsa-miR-19b-3p	30343695	6857599	hsa-miR-200c-3p	25757925
3385	hsa-miR-32-5p	29530052	36314	hsa-miR-141-3p	26025631
2767	hsa-miR-363-3p	25416050	119307	hsa-miR-181d-5p	Unconfirmed
5310940	hsa-miR-373-3p	Unconfirmed	3385	hsa-miR-620	Unconfirmed
3385	hsa-miR-576-5p	Unconfirmed	3385	hsa-miR-9-3p	Unconfirmed
36462	hsa-miR-21-5p	23834154	5310940	hsa-miR-128-3p	30890168
60750	hsa-miR-24-3p	Unconfirmed	60750	hsa-miR-29c-3p	29807360
60750	hsa-miR-205-5p	31602229	2767	hsa-miR-1236-3p	30805558
2767	hsa-miR-193b-3p	27918099	5310940	hsa-miR-660-5p	Unconfirmed
3385	hsa-miR-10a-5p	Unconfirmed	60750	hsa-miR-532-5p	Unconfirmed
3385	hsa-miR-33b-5p	Unconfirmed	31703	hsa-miR-18a-5p	Unconfirmed
3385	hsa-miR-376a-3p	Unconfirmed	3385	hsa-miR-431-5p	Unconfirmed
2520	hsa-miR-126-3p	Unconfirmed	5310940	hsa-miR-196a-5p	Unconfirmed

The CID of PubChem is used to indicate known MDIs in the RNAInter dataset. The first column records the top 1–25 MDIs. The second column records the top 26–50 MDIs. The evidence is indicated by the PubMed ID of the experimental literature.

Furthermore, to demonstrate the prediction ability for new drugs, we selected 5-fluorouracil (5-FU, CID:3385) as the investigated drug of the case study, which is a chemotherapy drug widely used in digestive system cancer and breast cancer (Wigmore et al., 2010). The MDIs related to 5-FU were removed from the dataset and the rest of MDIs were used to train the prediction model. Then we implemented the BNEMDI model to identify potential miRNAs that may interact with 5-FU. The top 50 predicted miRNAs are shown in Table 6. Among the top 10, 20, and 50 predicted miRNAs, there were 9, 17, and 37 miRNAs, which confirmed that they may interact with 5-FU by the previous literature.

**TABLE 6:** Top 50 associated miRNA of drug 5-FU predicted by BNEMDI.

Drug (CID)	miRNA	Evidence	Drug (CID)	miRNA	Evidence
3385	hsa-miR-21-5p	31918721	3385	hsa-miR-181b-5p	Unconfirmed
3385	hsa-miR-221-3p	27726102	3385	hsa-miR-26b-5p	30662808
3385	hsa-miR-126-3p	Unconfirmed	3385	hsa-miR-194-5p	30451820
3385	hsa-miR-200c-3p	28411308	3385	hsa-miR-103a-3p	27247088
3385	hsa-miR-222-3p	19956872	3385	hsa-miR-208a-3p	Unconfirmed
3385	hsa-let-7c-5p	33051247	3385	hsa-miR-18a-5p	32884453
3385	hsa-miR-214-3p	Unconfirmed	3385	hsa-miR-20b-5p	27878272
3385	hsa-miR-155-5p	30741544	3385	hsa-miR-663a	confirmed
3385	hsa-miR-93-5p	32426273	3385	hsa-miR-145-5p	32801865
3385	hsa-miR-18b-5p	25990502	3385	hsa-miR-24-3p	31646794
3385	hsa-miR-143-3p	19843160	3385	hsa-miR-19a-3p	24460313
3385	hsa-miR-181a-3p	29795190	3385	hsa-let-7a-5p	35071455
3385	hsa-miR-16-5p	18449891	3385	hsa-miR-4661-3p	Unconfirmed
3385	hsa-miR-27b-3p	24401318	3385	hsa-miR-27a-3p	24401318
3385	hsa-miR-107	26636340	3385	hsa-miR-200b-3p	32714549
3385	hsa-miR-34c-5p	Unconfirmed	3385	hsa-miR-9-5p	Unconfirmed
3385	hsa-miR-17-5p	32426273	3385	hsa-miR-101-3p	34086111
3385	hsa-miR-34a-5p	31802650	3385	hsa-miR-196a-5p	Unconfirmed
3385	hsa-miR-125b-5p	28176874	3385	hsa-miR-200a-3p	28496200
3385	hsa-miR-497-5p	26673620	3385	hsa-miR-802	Unconfirmed
3385	hsa-miR-29b-3p	34155879	3385	hsa-miR-197-3p	26055341
3385	hsa-miR-20a-5p	31760170	3385	hsa-miR-30b-5p	miR-30b
3385	hsa-miR-1915-3p	Unconfirmed	3385	hsa-miR-181b-2-3p	Unconfirmed
3385	hsa-miR-210-3p	31468617	3385	hsa-miR-100-5p	Unconfirmed
3385	hsa-miR-25-3p	35014676	3385	hsa-miR-153-3p	Unconfirmed

The CID of PubChem is used to indicate known MDIs in the RNAInter dataset. The first column records the top 1–25 MDIs. The second column records the top 26–50 MDIs. The evidence is indicated by the PubMed ID of the experimental literature.

For instance, Valeri et al. discovered that miRNA-21-5p, which ranks first in the top 50 predicted miRNAs can downregulate the expression level of human DNA MutS homolog 2 leading to 5-FU resistance in colon cancer patients (Liang et al., 2020). Moreover, the study proposed by Zhao et al. (2016) confirmed the overexpression of hsa-miR-221-3p will reduce the sensitivity of 5-FU and proved it can be a potential drug target for pancreatic cancer (Zhao et al., 2016). Moreover, through functional analysis, Jilek et al. (2020) demonstrated that has-let-7c-5p can elevate the exposure of 5-FU. They suggested that has-let-7c-5p and 5-FU can attenuate thymidylate synthase, which indicates that 5-FU can cooperate with has-let-7c-5p against hepatocellular carcinoma (Jilek et al., 2020). As stated before, this case study shows that BNEMDI can effectively find out the miRNAs interacting with given drugs.

## Conclusion

MDI prediction plays an important role in new drug target research. In this article, we proposed a novel computational model to predict unknown MDIs, namely, BNEMDI. We adopted a bipartite network embedding method BiNE to extract the topological feature from the MDI network. The chemical structure of drugs and the base sequence information of miRNAs are represented as the attribute feature by MACCS fingerprints and k-mer. When performed on five datasets (ncDR, RNAInter, SM2miR1, SM2miR2, and SM2miR3), BNEMDI gained average AUC values of 88.75, 87.23, 77.24, 79.92, and 81.86% under five-fold cross-validation, respectively. In addition, we experimented with other popular network embedding methods in different dimensions. Moreover, the case study on a common drug for cancer and all of the candidate miRNA–drug pairs demonstrated that the proposed model could be an effective tool for predicting MDI in real scenarios. The comprehensive results indicated that BNEMDI is a reliable and stable MDI predictor, economizing time and labor for drug target studies. Even so, the BNEMDI model possesses drawbacks. For new drugs and miRNAs, they are independent nodes in the bipartite network. The network embedding methods cannot learn any information from these independent nodes. Only attribute features can represent these nodes, and then the new interaction network can be updated according to the wet experimental results. In the future, we expect to seek more efficient network embedding methods and feature descriptors for mining the relationship between drugs and miRNAs.

## Data Availability

Publicly available datasets were analyzed in this study. These data can be found at: http://www.jianglab.cn/ncDR/index.jsp (ncDR) http://www.jianglab.cn/SM2miR/ (SM2miR) http://www.rnainter.org/ (RNAInter).
